# Multipolar radiofrequency ablation via three bipolar electrodes with C-arm type X-ray fluoroscopy assistance for hepatocellular carcinoma: An observational study

**DOI:** 10.1097/MD.0000000000030725

**Published:** 2022-09-23

**Authors:** Takashi Tanaka, Akira Anan, Kazuhide Takata, Hiromi Fukuda, Ryo Yamauchi, Shinjiro Inomata, Keiji Yokoyama, Yasuaki Takeyama, Satoshi Shakado, Shotaro Sakisaka, Fumihito Hirai

**Affiliations:** a Department of Gastroenterology, Faculty of Medicine, Fukuoka University, Fukuoka, Japan.

**Keywords:** C-arm type X-ray fluoroscopy, hepatocellular carcinoma, multipolar radiofrequency ablation, transcatheter arterial chemoembolization

## Abstract

The present study aimed to investigate the therapeutic efficacy and safety of the insertion technique of 3 bipolar electrodes in patients with hepatocellular carcinoma (HCC), using C-arm type X-ray fluoroscopy-assisted ultrasonography (US) in guiding a multipolar radiofrequency ablation (RFA) system. Seventy-three patients with HCC treated with a multipolar RFA system (1 electrode, n = 2; 2 electrodes, n = 56; 3 electrodes, n = 17) were enrolled in this retrospective cohort study. To analyze their therapeutic outcome in this study, we divided among 17 patients using 3 electrodes into 2 subgroups: the C-arm type X-ray fluoroscopy-assisted (n = 7) and the US-guided alone groups (n = 10). Therapeutic efficacy and safety were analyzed between the 2 groups. Multipolar RFA treatment was performed safely in all cases, and no severe adverse events occurred. Comparing the patient background of the group treated using 1 or 2 electrodes with that treated using 3 electrodes, larger-sized HCC was treated using 3 electrodes (*P* < .001). The differences in overall and recurrence-free survival rates between the 1- or 2-electrode and the 3-electrode groups were not significantly different (*P* = .843 and *P* = .891). Comparing the C-arm type X-ray fluoroscopy-assisted and the US-guided alone groups among patients treated using 3 electrodes, technical factors such as total ablation time and the number of sessions were not significantly different between the 2 groups. The local tumor progression rate was not significantly different between the 2 groups (*P* = .942). Multipolar RFA treatment was effective for the treating HCC; using 3 electrodes was suitable for larger-sized HCCs. The technical approach with C-arm type X-ray fluoroscopy assistance using 3 electrodes was useful for operators to perform safe and appropriate insertion techniques by synchronizing the US and X-ray fluoroscopy images.

## 1. Introduction

Radiofrequency ablation (RFA) is widely performed because it is easy, safe, cost-effective, and applicable as a minimally invasive technique for patients with hepatocellular carcinoma (HCC). RFA has favorable results comparable to those of liver resection.^[1–3]^ RFA systems for HCC involve 2 types of electrodes: monopolar and bipolar. The bipolar electrodes used in multipolar RFA systems have recently become available worldwide and enabled the acquisition of large ablative zones with a maximum diameter of 6.0 cm, referring to the “Dosimetry table,” which illustrates several patterns of shapes after the use of 1 to 3 internally cooled electrodes.^[4–6]^ However, the technique requires a parallel insertion using several electrodes, which is difficult to achieve using ultrasonography (US) guidance alone.

Biplane fluoroscopy-assisted RFA with transcatheter arterial chemoembolization (TACE) is technically feasible and effective for treating HCC.^[7–9]^ Furthermore, chemoembolization increases HCC visibility on fluoroscopy due to the accumulation of iodized oil. However, these studies only investigated the monopolar RFA system. To the best of our knowledge, the multipolar RFA system has not been studied yet.

The present study was aimed to assess the technical feasibility and effectiveness of multipolar RFA treatment especially when inserting 3 bipolar electrodes using C-arm type X-ray fluoroscopy assistance and US as guides, combined with TACE.

## 2. Materials and Methods

### 2.1. Ethics statement

This retrospective, single-center cohort study was approved by the local ethical review board (approval No. H20-10-003) of Fukuoka University Hospital. The study was conducted in accordance with the Declaration of Helsinki, and written informed consent was obtained from all patients before initiating therapy.

### 2.2. Study design and patient population

This retrospective cohort study included patients receiving treatment between April 2014 to March 2017 at Fukuoka University Hospital. During the study period, we enrolled 73 patients with HCC who underwent multipolar RFA as a curative locoregional treatment in our institute. We enrolled patients with HCC according to the following inclusion criteria: no more than 3 tumors with the largest tumor up to 5.0 cm in diameter; absence of tumoral invasion of the trunk or main portal veins; prothrombin INR of <1.5 and platelet count ≥50,000/mL; and cirrhosis of Child-Pugh class A or B. HCC was diagnosed for lesions that were hypervascular in the arterial phase and washed out in the portal venous or delayed phase using computed tomography (CT) and magnetic resonance imaging (MRI).

### 2.3. Transcatheter arterial chemoembolization

Several patients underwent TACE before multipolar RFA treatment. As previously reported, TACE was performed using the Seldinger technique and in a conventional technique.^[10,11]^ After performing diagnostic hepatic angiography, a 2.4 -Fr micro-catheter (Micro Ferret-18, William Cook, Bjaeverskov, Denmark) was selectively placed into the feeding arteries for selective embolization using a 0.014-inch micro-guidewire (Micromate guidewire, Terumo Clinical Supply, Gifu, Japan). After the hepatic artery was catheterized post-arteriography of the celiac and superior mesenteric vessels, TACE was performed on the hepatic artery that supplied the target tumor under super-selective catheterization using a micro-catheter; this was in accordance with blood distribution of HCC. An oil suspension was prepared using an emulsion of 2 to 5 mL of iodized oil (Lipiodol Ultra-Fluid, Gurbet Japan, Tokyo, Japan) and anticancer agents dissolved in contrast medium (Iopamiron 300 mgI/mL, Bayer Yakuhin, Osaka, Japan) at half the volume of iodized oil. The anticancer agent that included 10 to 30 mg epirubicin hydrochloride (Farmorubicin, Pfizer Japan, Tokyo, Japan) was used. The suspension was shaken by hand for a few minutes for better mixing just before being used. The iodized oil suspension was the tumor were filled (2–10 mL). Embolization was done according to tumor size and vascular diameter using 500 µm- to 1 mm porous gelatin sponge particles (Spongel, Astellas Pharma, Tokyo, Japan) from the feeding artery.

### 2.4. The multipolar RFA system procedure

Percutaneous RFA was performed by physicians with >5 years of experience in RFA treatment of liver tumors. RFA treatment was performed using a recently developed bipolar RFA system (CelonLabPOWER; OLYMPUS, Japan) in this study. This system has an operating frequency of 470 kHz and a maximum power output of 250 W. The bipolar electrodes had a diameter of 1.8 mm, active tip length of 20 or 30 or 40 mm (T20, T30, T40), and shaft length of 15 to 20 cm; they were internally cooled using a pump (Celon Aquaflow Ⅲ; OLYMPUS).^[4]^ We selected an active tip length approximating the tumor size; that is, 3 electrodes of 20 mm (T20–T20–T20) were selected for tumors 2.0 to 2.5 cm in diameter, 3 electrodes of 30 mm (T30–T30–T30) were selected for tumors 2.5 to 4.0 cm in diameter, and 3 electrodes of 40 mm (T40–T40–T40) were selected for tumors 4.0 to 5.0 cm in diameter. Electrodes were inserted into the liver, and a parallel or non-parallel (fan-shaped) pincer insertion technique was performed on the tumor edges. Radiofrequency energy was delivered using the generator’s impedance-based control algorithm. Overlapping ablations were applied depending on tumor size, shape, and location to achieve sufficient ablative zone margin (≥5 mm). At the end of the procedure, tract ablation was performed to prevent bleeding or tumor seeding. When required, artificial ascites were induced to improve the US window and decrease thermal injury to adjacent organs, including the diaphragm, lung, heart, gall bladder, stomach, and colon.

### 2.5. Multipolar RFA treatment with 3 bipolar electrodes using C-arm type X-ray fluoroscopy assistance

Of the 9 patients who underwent TACE, 7 underwent multipolar RFA with 3 bipolar electrodes assisted with a C-arm type X-ray fluoroscopy system (C-vision Safire17, Shimadzu Co., LTD. Kyoto, Japan) within 14 days after TACE. Whereas the C-arm angulation is right anterior oblique/left anterior oblique: 90/45 degrees, Craniomedial/Caudal: 35/35 degrees, and C-arm rotation around the patient’s longitudinal axis (which covered a 240° circular trajectory); the operator confirmed the position of the bipolar electrode in this angulation. US-guided imaging was used concurrently to determine a safe skin entry site, enable accurate targeting of the index tumor and avoid the traversal of critical structures, such as large vessels and other organs surrounding the liver. The patients were treated under local anesthesia with conscious sedation.

### 2.6. Clinical outcome

**Figure 1.**
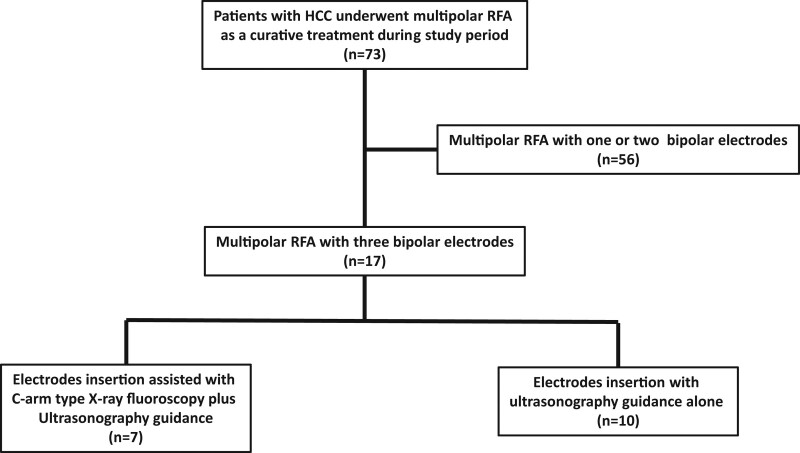
Patient selection process and the study groups.

We compared the efficacy of multipolar RFA treatment between the 1- or 2-electrode and the 3-electrode groups. Furthermore, we investigated the efficacy and safety of US-guided multipolar RFA treatment using 3 bipolar electrodes with C-arm type X-ray fluoroscopy assistance (Fig. 1).

### 2.7. Follow-up after ablation

The radiological response was assessed within 1 week after multipolar RFA treatment using contrast-enhanced dynamic CT or MRI. A tumor was considered completely ablated if no nodular or irregular enhancement adjacent to the ablative zone was visible in the arterial phase and if an ablative zone margin ≥5 mm from the edge of the tumor was observed in the portal phase. The latter also was defined as technical effectiveness.

Physicians examined the patients 4 weeks after RFA treatment, and liver function tests and tumor markers were measured once every 3 months. After the HCC eradication, recurrence was surveyed using contrast-enhanced dynamic CT or MRI every 3 months to detect early-stage local tumor progression after assessing the therapeutic effects of RFA.

### 2.8. Statistical analysis

The therapeutic efficacy of RFA was assessed based on standardization paper of image-guided tumor ablation.^[12]^ A comparison of baseline data between the 2 groups was conducted using Student *t* test for continuous variables and Fisher exact test for categorical variables. The overall survival, recurrence-free survival, and local progression rates were estimated using the Kaplan–Meier method. The differences in the local tumor progression rates between the 2 groups were compared using the log-rank test. A *P* value of <.05 was considered to be statistically significant. Statistical analyses were performed using JMP software for Windows, version 9.03 (SAS Institute, Cary, NC).

## 3. Results

### 3.1. Technical success and effectiveness compared between HCC patients treated with multipolar RFA using 1- or 2-electrode and 3-electrode.

**Figure 2.**
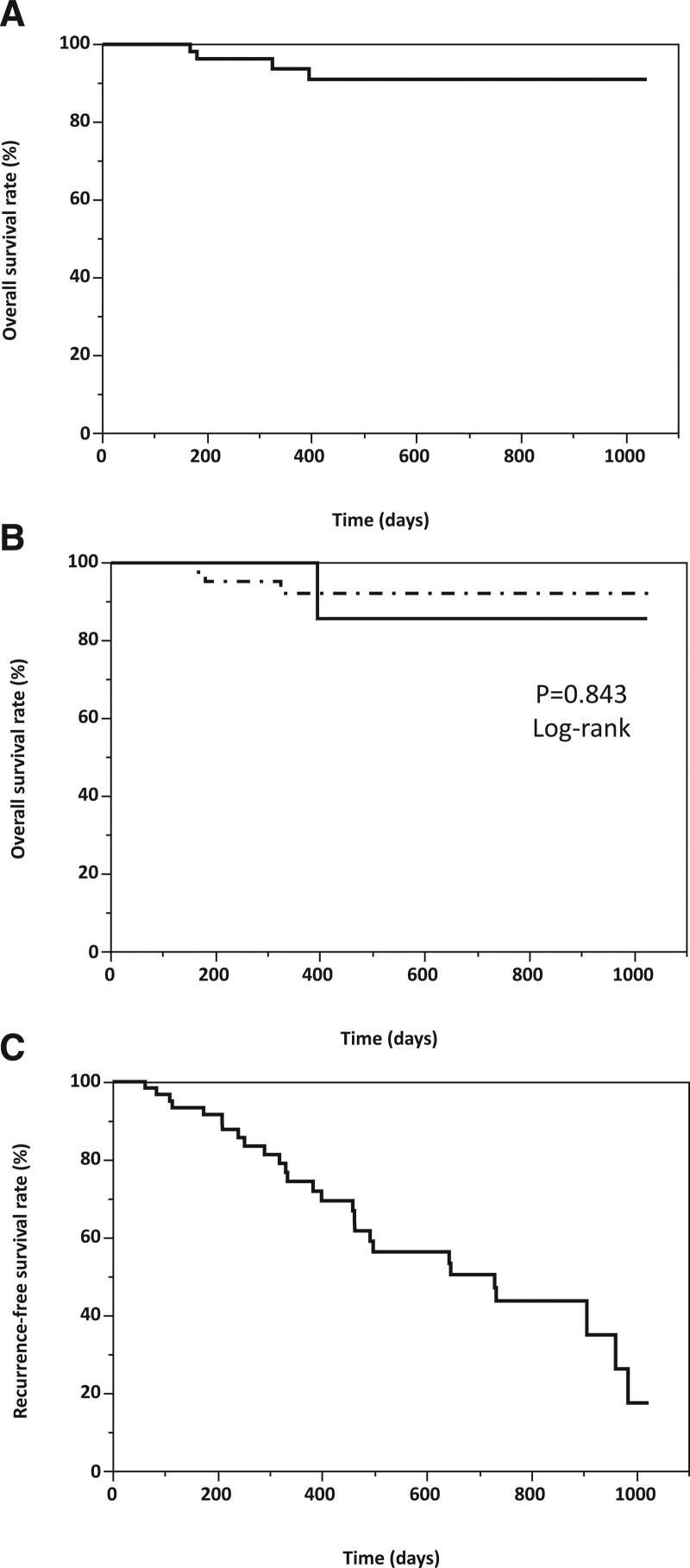
(A–D) Overall and recurrence-free survival rates in this study population. (A) Overall survival rate of the patient enrolled in this study (n = 73). (B) Overall survival rate between the group treated using 1 or 2 electrodes (n = 56; dashed line) and that treated using 3 electrodes (n = 17; solid line). (C) Recurrence-free survival rate of the patient enrolled in this study (n = 73). (D) Recurrence-free survival rate between the group treated using 1 or 2 electrodes (n = 56; dashed line) and that treated using 3 electrodes (n = 17; solid line).

Of the 73 patients with HCC who underwent bipolar RFA system 56 were treated using 1 or 2 electrodes (1 electrode; n = 2, 2 electrodes; n = 54) and 17 using 3 electrodes. The baseline characteristics of the patients enrolled in the study are shown in Table 1. The median tumor diameters in the 1- or 2-electrode and 3-electrode groups were 1.6 cm (range, 0.7–3.4) and 2.6 cm (2.0–4.5), respectively (*P* < .001). The number of tumors for which a sufficient ablative zone margin (≥5 mm) was acquired between the 1- or 2-electrode group and the 3-electrode groups was not statistically significantly different (*P* = .327). We analyzed overall and recurrence-free survivals in this study population. Kaplan–Meier curves for overall and recurrence-free survival rates are shown in Figure 2. The median overall survival was not evaluated (range, 50–1039); the overall survival rate at 1 and 2 years were 93.8% and 91% (Fig. 2A). Overall survival between the 1- or 2-electrode and 3-electrode groups was not significantly different (*P* = .843, log-rank) (Fig. 2B). Median recurrence-free survival was 730 days (range, 52–472), and the recurrence-free survival rate at 1 and 2 years were 74.4% and 43.7%, respectively (Fig. 2C). Recurrence-free survival between the 1- or 2- electrode and 3- electrode groups were not significantly different (*P* = .891, log-rank) (Fig. 2D).

**Table 1:** Patient backgrounds and tumor characteristics between treated using 1- or 2- electrode and 3- electrode groups.

Characteristics	1 or 2 electrodes group (n = 56)	3 electrodes group (n = 17)	*P* value
Age (yr)	72 (37-84)	75 (37-84)	.229
Gender (male/female)	35 / 21	8 / 9	.197
Child Pugh classification (A/B)	40 / 16	15 / 2	.137
Etiology HBV/ HCV/nBnC	7 / 36 / 13	4 / 9 / 4	.606
Tumor size (cm)	1.6 (0.7-3.4)	2.6 (2.0-4.5)	<.001
TACE + RFA (yes/no)	13 / 43	9 / 8	.023
Sufficient ablative zone (yes/no)	48 / 8	16 / 1	.327

Data are presented as number or median (range).

HBV = hepatitis B virus, HCV = hepatitis C virus, nBnC = non B non C, RFA = radiofrequency ablation, TACE = transcatheter arterial chemoembolization, US = ultrasonography.

### 3.2. Technical success and effectiveness among patients with HCC treated with multipolar RFA using C-arm type X-ray fluoroscopy assistance

During the study period, 7 consecutive patients with 7 HCC nodules met the inclusion criteria in our analysis (Table 2). The median diameter size of the nodules was 3.3 cm, with a range of 2.6 to 4.5 cm. All nodules were located in the right lobe of the liver, either directly in contacting the diaphragm (n = 3). All HCC nodules were ablated using 3 bipolar electrodes. Based on tumor size, the T30–T30–T30 pattern was used for 5 nodules, whereas the T40–T40–T40 pattern was used for 2 nodules. After TACE, all HCC nodules were clearly visible on fluoroscopy and were ablated with C-arm type X-ray fluoroscopy assistance. Four HCC nodules required only 1 ablation session, while 3 nodules required 2. The median total ablation time was 17 min 21 sec (range: 11 minutes 49 seconds to 24 minutes 20 seconds). Technical success with an ablative marginal zone ≥ 5 mm was achieved in all 7 nodules. Based on CT performed during 1-month follow-up, primary technical effectiveness was achieved in all 7 cases. There were no deaths related to the combined treatment, and major complications were not observed in this study. Local tumor progression was observed in only 1 case during the follow-up period (median: 18 months; range: 3–52 months). The patient with local tumor progression underwent additional RFA treatment after recurrence.

**Table 2:** Characteristics of hepatocellular carcinoma treated with multipolar RFA system with C-arm type X-ray fluoroscopic assistance, and treatment outcomes.

Patient no	Age	Gender	Etiology	CP	AFP (ng/mL)	DCP (mAU/mL)	Location	Liver dome location	Size (cm)	Combination of bipolar electrodes	Number of sessions	Total ablation time	Technical effectiveness	AEs
1	63	M	HCV	A	16.6	42	S8	No	2.6	T30-T30-T30	1	11m49s	Yes	n
2	77	F	HCV	A	38.9	1529	S5	No	2.7	T30-T30-T30	2	24m20s	Yes	PE
3	74	F	HCV	A	3.8	82	S7	Yes	4.3	T40-T40-T40	1	16m33s	Yes	PE
4	78	F	HBV	A	2.7	319	S7	No	3.3	T30-T30-T30	1	13m40s	Yes	n
5	77	M	HCV	A	1404	37	S8	Yes	4.5	T40-T40-T40	1	17m21s	Yes	PE
6	68	F	HCV	A	2.3	38	S8	No	3.3	T30-T30-T30	2	22m54s	Yes	n
7	71	M	HBV	A	N.E	N.E	S7	Yes	3.5	T30-T30-T30	2	17m53s	Yes	n

AEs = adverse events, AFP = alpha-fetoprotein, CP = Child-Pugh classification, DCP = des-γ-carboxy prothrombin, HBV = hepatitis B virus, HCV = hepatitis C virus, N.E = not examination, PE = pleural effusion, RFA = radiofrequency ablation, TACE = transcatheter arterial chemoembolization.

**Figure 3.**
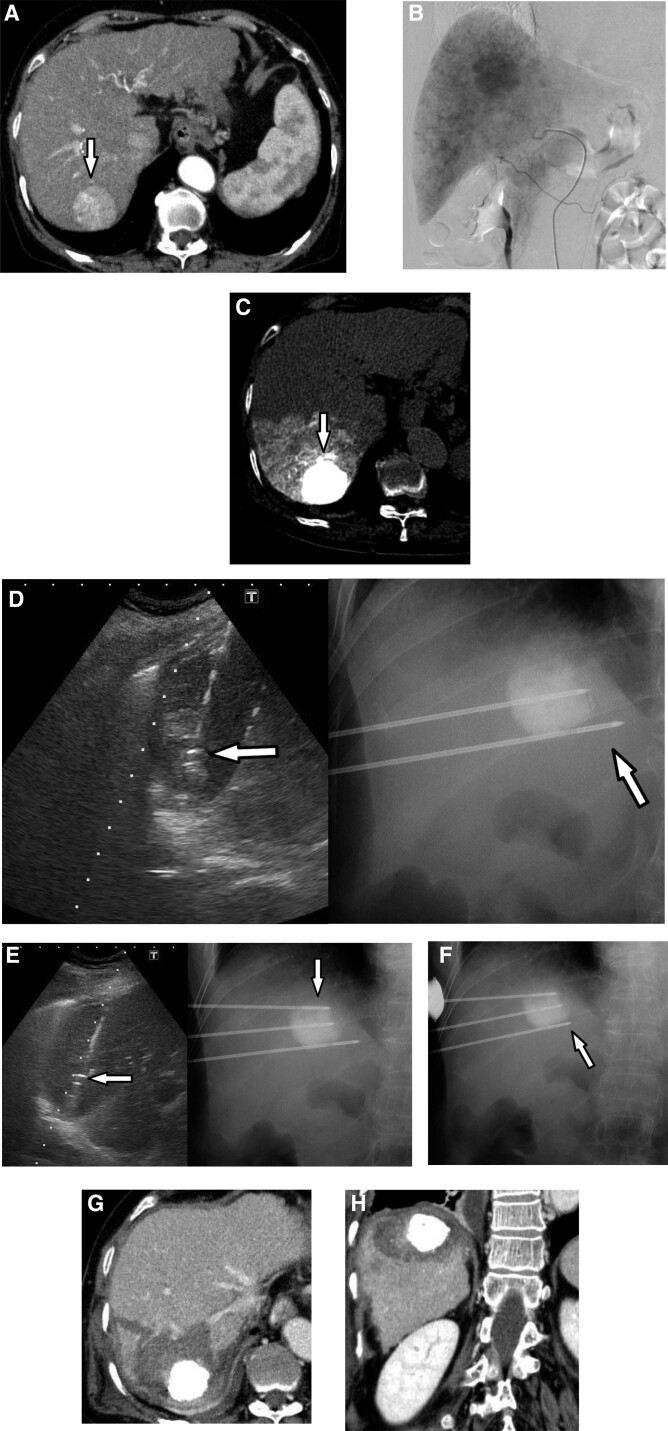
(A–H) Multipolar RFA treatment assisted with C-arm X-ray fluoroscopy imaging. A 74-year-old female patient with 4.3 cm right-lobar HCC (Segment 7). Twelve days post-TACE, multipolar RFA using 3 bipolar electrodes was performed. (A) The tumor shows hypervascularity in the early phase with dynamic CT imaging (white arrow). (B) Angiography and TACE was performed. (C) Remarkable epirubicin plus lipiodol emulsion was accumulated into the tumor (white arrow). (D, and E) Since the tumor outline is clear on fluoroscopy images, safe and appropriate insertion is achieved by synchronizing the US and fluoroscopic image (white arrows). (F) Immediately before ablation, the electrode is easily adjustable via reflecting fluoroscopic image (white arrow). Superiority over CT-guided RFA implies achieving proper insertion by real-time synchronization of the US and X-ray fluoroscopy images. (G and H) Portal venous phase axial and coronal CT images 3 days post-RFA show HCC with iodized oil retention and non-enhancing peri-tumor area, indicating technical success. CT = computed tomography, HCC = hepatocellular carcinoma, TACE = Transcatheter arterial chemoembolization, RFA = Radiofrequency ablation, US = ultrasonography.

In multipolar RFA, 3 electrodes converged on the tumor, and the equidistant alignment of the needle tips is the most crucial factor in acquiring the ideal shape of the ablative zone and sufficient ablative margin (≥5 mm). In the case shown in Figure 3, we modified 1 needle’s tip in real-time with C-arm type X-ray fluoroscopy assistance, permitting the acquisition of the targeted ablative zone volume and sufficient ablative margins in 1 session. If 1 needle tip is farther or more advanced than the 2, the ablative zone may be irregular, leading to an insufficient therapeutic effect. Furthermore, with slight restrictions, the C-arm function was useful in helping the operator to visualize each electrode’s position by changing the angles on the X-ray fluoroscopy images.

### 3.3. Background and therapeutic efficacy of multipolar RFA treatment between US-guided alone group and C-arm type X-ray fluoroscopy assistance group

**Figure 4.**
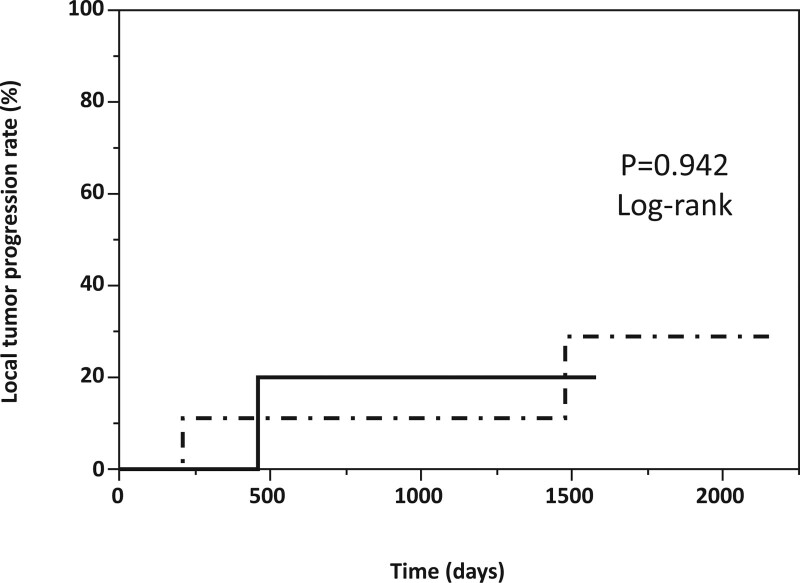
Local tumor progression rates between the US-guided alone (n = 10; dashed line) and C-arm type X-ray fluoroscopy assistance groups (n = 7; solid line). The cumulative local tumor progression rates between the US-guided alone and C-arm type X-ray fluoroscopy assistance groups were not significantly different. US = ultrasonography

The number of patients with HCC treated using a multipolar RFA system with 3 bipolar electrodes was 17 in our institute. Ten patients underwent multipolar RFA with US-guided alone, and 7 received C-arm type X-ray fluoroscopy assistance. The baseline characteristics of the patients in each group enrolled in this study are shown in Table 3. The median tumor diameters between US-guidance alone and C-arm type X-ray fluoroscopy groups were 2.5 cm (2.0–3.8 cm) and 3.3 cm (2.6–4.5 cm), respectively (*P* = .025). Although all patients underwent TACE before multipolar RFA treatment among the C-arm type X-ray fluoroscopy group, TACE was performed in only 2 cases in the US-guided alone group (*P* = .001). Technical features, such as total ablation time, number of sessions, and therapeutic efficacy, including acquired sufficient ablative margin (≥5 mm), were not statistically different between the 2 groups. The cumulative local tumor progression rates between the US-guided alone and C-arm type X-ray fluoroscopy assistance groups were not significantly different (*P* = .942) (Fig. 4).

**Table 3:** Patient backgrounds and tumor characteristics between US-guidance alone and C-arm type X-ray fluoroscopy groups.

Characteristics	US-guidance alone (n = 10)	C-arm X-ray fluoroscopy (n = 7)	*P* value
Age (yr)	77.5 (37-84)	74 (63-78)	.867
Gender (male/female)	5 / 5	3 / 4	.772
Child Pugh classification (A/B)	8 / 2	7 / 0	.208
Etiology HBV/ HCV/ nBnC	2 / 4 / 4	2 / 5 / 0	.102
Tumor size (cm)	2.5 (2.0-3.8)	3.3 (2.6-4.5)	.025
APF (ng/mL)	5.2 (2.3-123)	3.8 (2.0-1404)	.388
DCP (mAU/mL)	20 (14-897)	38 (15-319)	.865
TACE + RFA (yes/no)	2 / 8	7 / 0	.001
Sufficient ablative zone (yes/no)	7 / 1	7 / 0	.919
Number of RFA session (1 / 2 / 3)	5 / 4 / 1	4 / 3 / 0	.575
Total ablation time	14m51s (12m4s-41m16s)	17m21s (11m49s-24m20s)	.778

Data are presented as number or median (range).

AFP = alpha-fetoprotein, DCP = des-γ-carboxy prothrombin, HBV = hepatitis B virus, HCV = hepatitis C virus, nBnC = non B non C, RFA = radiofrequency ablation, TACE = transcatheter arterial chemoembolization, US = ultrasonography.

### 3.4. Procedural complication

**Figure 5:**
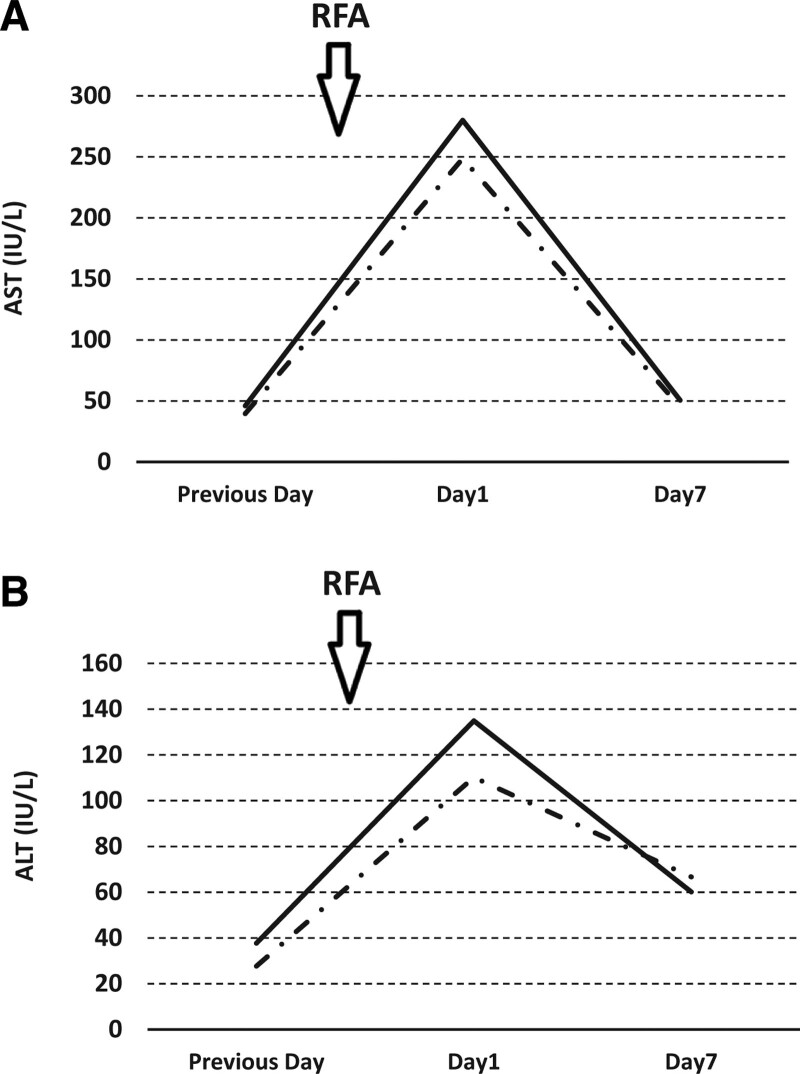
. (A) Change in aspartate aminotransferase (AST) levels between US-guided alone (n = 10; dashed line) and C-arm type X-ray fluoroscopy assistance groups (n = 7; solid line) before treatment, 1 and 7 days after the treatment. (B) Change in alanine aminotransferase (ALT) levels between US-guided alone (n = 10; dashed line) and C-arm type X-ray fluoroscopy assistance groups (n = 7; solid line) before treatment, 1 and 7 days after the treatment. US = ultrasonography.

Among the C-arm type X-ray fluoroscopy group, multipolar RFA therapy was performed safely in all cases, and severe adverse events did not occur. Although moderate pleural effusion had appeared in 3 patients just after the RFA procedure, no patients experienced dyspnea and all recovered 1 month after RFA treatment. Furthermore, we investigated the change in aspartate aminotransferase and alanine aminotransferase levels after the multipolar RFA treatment. We found that the change in aspartate aminotransferase/alanine aminotransferase levels between the US-guided alone and the C-arm type X-ray fluoroscopy assistance groups was not significantly different at 1 and 7 days after the treatment (Fig. 5).

## 4. Discussion

Several studies have reported that multipolar RFA is effective for treating HCC. Kawamura et al demonstrated that the no-touch insertion technique is useful for preventing tumor recurrence within the same liver segment after RFA in patients with HCC.^[13]^ Ptiti et al reported that multipolar RFA is a safe and effective treatment for subcapsular HCC not puncturable via the non-tumoral liver parenchyma.^[14]^ Lin et al reported that multipolar RFA can effectively treat HCCs sized 3.1–7.0 cm with a comparable outcome between medium- and large-sized tumors and among the Barcelona Clinic Liver Cancer group stages A to B2.^[15]^ In this study, we investigated overall and recurrence-free survivals between the 1- or 2-electrode and the 3-electrode groups. We found that the 2 groups’ overall and recurrence-free survival rates were not significantly different. However, the median tumor diameter between the 2 groups was significantly different (1.6 cm vs 2.6 cm, *P* < .001); treatment using 3 electrodes procedure was suitable for larger tumor ablation ≥2.0 cm in diameter.

However, the technique required a parallel insertion with several electrodes, which is difficult using US-guided imaging alone particularly when using 3 electrodes. Hence, the CT-guided assistance insertion technique was often reported in multipolar RFA treatment.^[6]^ Hirooka et al reported using ultrasonographic 3-dimensional images in new simulator system (3D-SIM Navigator; Hitachi Healthcare, Tokyo, Japan), which simulates cautery shape from the number of bipolar electrodes with US to assist insertion technique for several bipolar electrodes aligned equidistantly.^[16,17]^ Using X-ray fluoroscopy assistance in RFA treatment for patients with HCC combined with TACE is effective as treatment support for sites difficult to treat with conventional US-guided techniques.^[7–9]^ Therefore, we considered that C-arm type X-ray fluoroscopy assistance might be effective for multipolar RFA treatment, particularly during the insertion technique using 3 electrodes. To the best of our knowledge, this is the first report of multipolar RFA treatment with C-arm type X-ray fluoroscopy assistance for patients with HCC.

Our study yielded more benefits than expected. The ability of the C-arm type X-ray fluoroscopy to confirm how each bipolar electrode is heading toward the tumor with lipiodol in real-time, when applied in combination with RFA treatment, is quite useful. It can also visualize the positional relationship between the 3 bipolar electrodes, which is difficult to appreciate on US images alone. The real-time operation was a major advantage over CT-guided RFA treatment. Correcting the tips of the electrodes according to the position of the other bipolar electrodes was made possible and easy (Fig. 3 and Supplementary Video, Supplemental Digital Content, http://links.lww.com/MD/H386).

In this study, we obtained the ideal shape of the ablative zone using C-arm type X-ray fluoroscopy assistance. Frericks et al reported that among 18 patients with HCC treated with the multipolar RFA system, 13 (72%) had a concentric configuration without substantial shape deformities, 2 ablation zones (11%) were moderately eccentric, and 2 (11%) were severely eccentric.^[4]^ These deformities may result from perfusion, with an incomplete fusion of the ablative zones surrounding the individual probes, and was regarded as a consequence of the technical process. Therefore, C-arm type X-ray fluoroscopy assistance is useful for multipolar RFA treatment, particularly in several electrode insertion techniques, in acquiring the ideal shape of the ablative zone.

We compared the case of multipolar RFA using 3 electrodes with US-guided alone (n = 10) to evaluate the superiority in technical aspects and therapeutic effect of C-arm type X-ray fluoroscopy assistance. However, 2 groups had no significant difference in technical features, such as total ablation time and number of ablation sessions. Regarding therapeutic effect, local tumor progression was observed in 1 patient in the C-arm type X-ray fluoroscopy assistance group and 2 in the US-guided alone group. Since there was no significant difference in the local tumor progression rates with the log-rank test between the 2 groups (*P* = .942), we concluded additional cases are necessary to evaluate superiority with C-arm type X-ray fluoroscopy assistance and to clarify long-term therapeutic outcomes.

Our study has several limitations. First, the study design was retrospective and non-comparative. Furthermore, the number of cases was smaller than those reported in previous studies on RFA systems for the treating HCC with X-ray fluoroscopy assistance. Because the multiple electrode insertion techniques were difficult in areas adjacent to other organs or near the vessels in the left lobe, all tumors treated with this procedure were existed in the right lobe, which was why the sample size was small. Second, patients with HCC who had complications with contrast-enhanced agents due to moderate to severe renal dysfunction (except end-stage renal disease on hemodialysis) or allergy could not undergo TACE and they could not receive multipolar RFA treatment with X-ray fluoroscopy assistance.

In conclusion, the novel approach presented in this study may help operators of multipolar RFA therapy for HCC, particularly those using 3 bipolar electrodes, to easily visualize the positional relationship between individual electrodes around the tumor site. This approach can also help achieve safe and appropriate insertion by synchronizing US and C-arm type X-ray fluoroscopy images. Examining more cases is necessary to verify the effectiveness and safety of this procedure.

## Acknowledgments

We appreciate all of the patients who participated in this study and their families. We are grateful as well to all the investigators, physicians, nurses, radiologic technologists and radiologists who helped us with this study. We would like to thank Editage (www.editage.com) for English language editing.

## Author contributions

**Conceptualization:** Takashi Tanaka.

**Data curation:** Takashi Tanaka, Kazuhide Takata, Hiromi Fukuda, Ryo Yamauchi, Shinjiro Inomata, Keiji Yokoyama, Yasuaki Takeyama, Shotaro Sakisaka.

**Formal analysis:** Takashi Tanaka, Akira Anan, Satoshi Shakado, Fumihito Hirai.

**Investigation:** Takashi Tanaka.

**Methodology:** Takashi Tanaka, Akira Anan, Kazuhide Takata.

**Project administration:** Fumihito Hirai.

**Supervision:** Fumihito Hirai.

**Writing – original draft:** Takashi Tanaka.

**Writing – review & editing:** Takashi Tanaka, Satoshi Shakado, Fumihito Hirai.

## Correction

Figures 2 and 3 were swapped when originally published and have since been corrected.
